# The experiences of patients with diabetes and strategies for their management during the first COVID-19 lockdown: a qualitative study

**DOI:** 10.1186/s12912-022-00911-4

**Published:** 2022-05-24

**Authors:** Mireia Vilafranca Cartagena, Glòria Tort-Nasarre, Maria Romeu-Labayen, Josep Vidal-Alaball

**Affiliations:** 1 Department of Nursing, Faculty of Health Science and Welfare, University of Vic-Central University of Catalonia (UVIC-UCC), Av. Universitaria 4-6, 08242 Manresa, Spain; 2 Althaia Fundation, C/Dr Joan Soler 1-3, 08243 Manresa, Spain; 3 Department of Nursing, Faculty of Nursing and Physiotherapy, University of Lleida, C/Montserrat Roig, 25198 Lleida, Spain; 4 SAP ANOIA. Gerencia Territorial Catalunya Central. Institut Català de La Salut, 087272 Sant Fruitós del Bages, Spain; 5 AFIN Research Group and Outreach Centre, Autonomous University of Barcelona. Campus Bellaterra, Cerdanyola del Vallès, Spain; 6 Department of Public Health, Mental Health and Mother-Infant Nursing, Faculty of Medicine and Health Sciences, University of Barcelona, L’Hospitalet del Llobregat, Spain; 7 Health Promotion in Rural Areas Research Group, Gerència Territorial de La Catalunya Central, Institut Català de La Salut, 08272 Sant Fruitós de Bages, Spain; 8 Unitat de Suport a La Recerca de La Catalunya Central, Fundació Institut Universitari Per a La Recerca a L’Atenció Primària de Salut Jordi Gol I Gurina, 08272 Sant Fruitós de Bages, Spain

**Keywords:** COVID-19, Type 2 diabetes mellitus, Patient isolation, Nurses, Primary health care, Qualitative Research

## Abstract

**Background:**

During the pandemic, primary care systems prioritised attention to COVID-19 patients; chronically ill patients, such as people with Type 2 Diabetes were obliged to take more responsibility for their own care. We aimed to analyse the experiences of patients with Type 2 Diabetes Mellitus during the stay-at-home order that was in place during the first wave of the COVID-19 pandemic and identify the strategies and resources used in managing their care.

**Method:**

We conducted a qualitative descriptive study. The participants were ten patients with type 2 Diabetes Mellitus who experienced strict lockdown during the first wave of the COVID-19 pandemic in Catalonia, Spain, selected using intentional sampling. We recorded semi-structured interviews with the participants and conducted thematic analysis.

**Results:**

We identified 14 subthemes, which we then grouped into three overarching themes: 1) anxiety, fear, and vulnerability (anxiety, fear, vulnerability, rethinking life, loneliness, sadness), 2) insufficient diabetes monitoring by the health system (health care received, glycaemic control, view of treatment by health providers) and proactive self-care (changes in daily routine, diet, physical activity, medication, personal protective equipment & social distancing).

**Conclusion:**

Despite the exceptional nature of the situation and the stress, worry, and changes in their daily lives, many respondents reported that they had successfully modified their lifestyles. Self-care was effective during confinement and was based on a process of adaptation using the resources available, without face-to-face contact with primary care health staff.

**Relevance to clinical practice:**

These results can help to guide the design and implementation of self-care-focused strategies and also to explore new ways of empowering patients without access to health care personnel.

## Introduction

Novel coronavirus disease (COVID-19) is a highly transmissible, rapidly spreading disease which has had a dramatic impact all over the globe. Although the overall mortality rate due to COVID-19 is relatively low [1], diabetes has emerged as a prominent comorbidity, associated with a severe and acute picture of respiratory distress and increased mortality. Thus, patients with chronic diseases such as type 2 diabetes (T2D) appear to be particularly vulnerable to the effects of the virus, and T2D is a major risk factor for poor prognosis in COVID-19 infection [2].

With the outbreak of the pandemic, governments imposed policies to reduce the transmission of the virus, including quarantine, isolation, social distancing and stay-at-home orders. These exceptional measures had a direct effect on the health behaviours of patients with chronic pathologies such as T2D [3]. Many patients with diabetes have encountered barriers to care due to the policies introduced to combat COVID-19, although maintaining good blood glucose control in these patients has proved to be an effective measure in preventing the transmission of the virus [4].

## Background

Type 2 diabetes cannot be cured, but lifestyle changes such as following a healthy diet, regular physical activity, and maintaining normal body weight can slow the progression of the disease and reverse its effects [5]. However, previous studies have shown that long-term maintenance of weight loss and complete adherence to diet and physical exercise recommendations is rare, especially in the adult population. Understandably, during the pandemic, many patients with T2D have found it particularly difficult to adhere to these lifestyle recommendations due to the restrictions on their access to health services and the problems in obtaining fresh food and in exercising [6].

In Catalonia (Spain), a stay-at-home order took place during the first wave of COVID-19, March 14 to May 2, 2020, at which time the measures were progressively relaxed. Stay-at-home orders (or “lockdown”) are implemented when quarantine for exposed patients and isolation for infected patients are insufficient to contain the spread of a disease [7]. During the seven weeks of strict lockdown in Spain, people were only allowed to leave home to receive medical treatment, buy food or work as an essential worker. Leaving home for exercise was prohibited, and non-essential businesses were shuttered.

Early research shows mixed effects of COVID-19 lockdowns on patients with diabetes. [8]) show that while glucose levels for type 1 diabetes patients improved significantly, those for T2D worsened in the short term. Makki et al. [9] show that patients with T2D had better glycaemic control during lockdown, but they do not specify whether the lockdown conditions were as strict as those in Spain.

Nursing professionals have a vital role to play in educating patients about the need to adapt their lifestyles and in helping them to modify their behaviour with respect to their health [10]. During the pandemic, primary care nurses have been obliged to prioritise care for COVID-19 patients [11], and as a result they have had to postpone the care of the chronically ill [12]. In this scenario, innovative strategies are needed to monitor and motivate diabetic patients who have had to take on more responsibility for their care [13].

Qualitative research on the experience of patients with COVID-19 has provided valuable information [4]. However, few qualitative studies have addressed the experiences of patients with chronic pathologies during the pandemic, and even fewer in patients with T2D. People with chronic conditions experienced a confluence of the COVID-19 pandemic and chronic diseases in the context of difficulty in accessing healthcare, sedentary lifestyle and increased stress and anxiety [14]. Shi et al. describes the perceived barriers to diabetes self-management of people with T2D during the pandemic: inadequate knowledge and behavioural beliefs, shortage of resources, health problems, negative emotions and lack of support [15]. A structured analysis of the experiences of these patients would provide a valuable tool for organising the community and human resources needed in similar situations.

The aim of the present study is to analyse the experiences of patients with T2D that were under a stay-at-home order during the first wave of the COVID-19 pandemic and to identify the strategies and resources used in the management of T2D in this new situation.

## Methods

### Design

We conducted a qualitative descriptive study, a design that is suited to arriving at a deeper understanding of practice in applied disciplines and is especially pertinent when the goal is to understand participants’ perspective and experience [16]. We began with a deductive approach to develop the interview guide and then conducted an inductive analysis of the resulting data. The study is part of an ongoing project about diabetes and physical activity, which was underway when the pandemic began (Authors, in progress).

### Participants

Sampling was intentional [17]. The participants were the ten patients with T2D from four different primary health centres in central Catalonia (Spain) that were participating in our ongoing study about diabetes and physical activity. The inclusion criteria were adults aged 55 to 79 years diagnosed with T2D at least two years previously. We chose this age range because 55 is the age at which the prevalence of T2D begins to increase rapidly in the population, and a cut-off at 79 allowed us to ensure that participants were young enough to conduct physical activity [18]). Additional inclusion criteria were having no complications associated with T2D, having good metabolic control (hbA1c < 7), and showing good adherence to T2D treatment (defined as adherence to prescribed medication for T2D, physical activity > 150 min/week, and healthy diet). The exclusion criteria were gestational diabetes or type 1 diabetes, cognitive impairment, or admission to hospital during confinement. All ten participants from our initial study agreed to a follow-up interview about their experiences of COVID-19. Data saturation [19] was reached by the tenth interview, when we detected that no relevant new information was emerging.

### Data collection

Data were collected through a semi-structured interview. The research team developed a set of interview questions relevant to the study objectives, based on the researchers’ clinical experience and a review of the scarce existing literature about patients with chronic illness during the pandemic: How is the COVID-19 pandemic affecting you as a person with diabetes? Can you describe the effect of the stay-at-home order on you at a personal, family, and professional level? Describe to me the care you received for your T2D during the stay-at-home order. How did your lifestyle change (In what sense? Can you tell me?). During the interview, follow-up questions were asked to encourage participants to provide additional details about their perspective.

The interviews were conducted by the principal investigator (PI) between July 2020 and January 2021. In the initial interviews for the ongoing study about T2D and physical activity, the PI had conducted interviews with the participants lasting approximately 45 min. When the pandemic broke out, the team devised a second phase of the study, and the PI invited the participants to a follow-up interview about their COVID-19 experiences. All ten agreed to participate and gave their informed consent. We opted for telephone interviews because we thought it would be easier for participants than video conferencing. We suggested that participants conduct the interview from a quiet place in which they wouldn’t be interrupted. This second interview lasted between 15 and 35 min, meaning that for each participant we have a total of between 60 and 80 min of recorded data. Participants’ confidentiality was protected by giving them pseudonyms. The voice files and transcriptions were encrypted and stored on a computer protected with an encrypted password. The interviews were performed and transcribed in Catalan or Spanish, depending on the preference of the participant. Later, the transcribed interviews were returned to participants for their approval. All participants accepted their transcribed interviews without changes.

### Data analysis

Data were analysed using thematic analysis [20] by ATLAS ti ®vs 9 support. We identified and reported patterns that emerged from the data and arranged them systematically to shed light on the research questions, while trying to keep faithful to the perspectives expressed by participants [16]. We conducted the analysis in the following phases:

Phase 1 Become familiar with the data by listening to recordings, transcribing them, and reading and rereading the transcripts. Entering transcripts into software Atlas-ti vs 9. Author 1 (MCV) participated in this phase.

Phase 2: Segmenting the meaning units in the transcripts and inductively grouped them to create subthemes and identify relationships among them. Author 1 participated in this phase.

Phase 3: Group the meaning units and abstracted the subthemes. Define the parameters of each subtopic. 14 subtopics have been tagged. Author 1 participated in this phase.

Phase 4: Group the subthemes into overarching themes (which became the primary structure for our analysis). Which in turn we grouped into three themes. Devise a glossary of themes. Author 1 participated in this phase.

Phase 5: Revised, discussed and agreed upon the subthemes and themes while returning to the data to verify the analysis. Authors 1, 2 (MRL) and 4 (GTN) participated in this phase.

Phase 6: Write the research report. Authors 1, 2 and 4 participated in this phase. Author 3 (JVA) examined both the processing and product of the research study.

### Rigour, reflexivity and quality criteria

The trustworthiness of data was determined by Credibility, Dependability, Conformability, Transferability [21].

Credibility has been achieved thanks to the analyst triangulation, to undertook constant revisions of the themes, subthemes and units of analysis and evaluation, ensuring qualitative validity by authors 1, 2 and 4. Transferability has been achieved by describing a phenomenon in sufficient detail to transferable to other settings and people. Dependability was ensured in this study thanks to the review by the third researcher who examined both the processing and product of the research study. Confirmability was achieved through the reflective effort of each researcher to be aware of and try to limit the influence of their own positionality on their analysis. As well as a transparent description of the research steps taken from the start of a research project. All methods were carried out by relevant guidelines and regulations.

The research team have experience with qualitative research and resolved disagreements by consensus, and complied with the Consolidated Criteria for Reporting Qualitative Research [22].

## Results

Ten patients with T2D from four primary care centres in central Catalonia (Spain) participated in the study. Table 1 displays the participants’ main sociodemographic characteristics. Ages ranged from 58 to 79 years, and 60% of participants had had T2D for more than 10 years; most also had a past history of pathology other than T2D.

**Table 1:** Sociodemographic characteristics of the participants

Patient	Age	Gender	Level of schooling	Employment status	Years of evolution of T2D	Relevant medical history	Marital status (widowed, married)/ living with a partner	HbA1c	Treatment
Patient 1	69	Female	Compulsory education	Retired	18	T2D, obesity, HBP, lumbar arthrosis	Married/living with partner	7	Insulin
Patient 2	76	Male	Compulsory education	Retired	5 (after kidney transplant)	T2D, HBP, mechanical aortic prosthesis, permanent atrial fibrillation, kidney transplant	Married/living with partner	6.2	Oral antidiabetic
Patient 3	70	Female	Compulsory education	Retired	7	T2D, breast cancer, HBP, right knee prosthesis	Married/living with partner	7	Oral antidiabetic
Patient 4	60	Female	Compulsory education	Retired	11	T2D	Married/living with partner	6.9	Oral antidiabetic
Patient 5	62	Female	University degree	Employed	16	T2D, hypothyroidism and Brugada syndrome	Married/living with partner	6.9	Oral antidiabetic and insulin
Patient 6	67	Male	Compulsory education	Retired	8	T2D	Married/living with partner	7	Oral antidiabetic
Patient 7	69	Male	Compulsory education	Retired	10	T2D, CPOD, HBP	Widowed/living with daughter	6.7	Oral antidiabetic and insulin
Patient 8	58	Male	Secondary education	Employed	4	T2D, ex-smoker (17 years ago), anxiety, HBP	Unmarried/lives alone	5.5	Oral antidiabetic
Patient 9	79	Male	Compulsory education	Retired	15	T2D, HBP	Married/living with partner	7	Oral antidiabetic
Patient 10	70	Male	University degree	Retired	11	T2D, HBP, generalised seizure in 2014	Unmarried/lives alone	5.7	Oral antidiabetic

*T2D* type 2 diabetes, *HBP* high blood pressure, *HbA1c* Hemoglobulin A1c test

In our inductive analysis, we identified 14 subthemes, which we grouped into three themes: 1) anxiety, fear and vulnerability, 2) insufficient diabetes monitoring by the health system, and 3) proactive self-care. Table 2 shows an example of the final themes, the codes from which they are built, and an example of a meaning unit from each code.

**Table 2:** Themes and codes

Theme	Code	Example
1. Anxiety, fear, and vulnerability	Anxiety	3:10 (P3) You can’t live with this anxiety…. We have enough with other things that are going on…
	Fear	2:6 (P2) I have… because as far as what you can see on TV… and also they tell you that people who get it have a hard time. And at my age… which is pretty old
	Rethinking life	10:5 (P10) Time for yourself in silence. Whether you like it or not… and of course you have time for lots of things. I mean, to remember, to analyse behaviours about who knows what and when, to rethink a lot of things…
	Loneliness	8:9 (P8) Well I’ve felt alone
	Sadness	7:15 (P6) The only thing that made me a bit sad is that I was used to seeing my grandson who lives in Vic. I went up every 8 or 10 days, and obviously that made me a bit sad
	Vulnerability	8:16 (P8) I’m a person that in theory has diabetes and high blood pressure… (silence) (…). I… it was more probable that I would have the disease
2. Insufficient diabetes monitoring by the health system	Health care received	1:9 (P1) With my nurse I have a close relationship, and I say, “Listen, what’s going on? Look, I’ve been watching my sugar and things”. And she says that we’re overwhelmed, overwhelmed. I had to go buy materials and I say, “So, have you found a place [for me to buy them]?” and she says, “Ok, right now I’ll look for it; I have a minute”. I mean, she did everything for me
	Glycaemic control	7:13 (P7) When we were in lockdown, I checked my sugar, and it was at 100. If it had been at 200 or 250 then I would have worried. But since it was never over 130. I check it in the morning. For a diabetic that’s perfect, for glucose
	View of treatment by health provides	1:13 (P1) I tried to take good care of myself so that I wouldn’t have to go running [to the health centre] or anything, but yeah, a lot of things were missing. And even now when they have to go to the homes of people who can’t go to the health centre, which seems really good to me, but they can’t do everything
3. Proactive self-care	Changes in daily routine	9:4 (P9) Before I walked, I went to buy bread, I went out to walk, and then I went to the Generalitat with some friends, and we played cards. We spent the morning until 12 or 12:30 pm. Then I came home, I had lunch, I went out to take a walk. But not during the lockdown
	Diet	11:7 (P1) No, my diet was the same. The only thing is that since you’re at home, it seems like you snack more. I gained weight from snacking more. Of course! You know what? The days seemed loooong to me. So, I ate a little bit, now have an afternoon snack. Ok… an hour has gone by, now I’ll eat that…
	Physical activity	7:7 (P7) The only thing I did was to walk inside my home. I went to the bedroom, from one wall to the other. And I entertained myself a little bit that way, because I knew it was the only physical activity I could do then
	Medication	13:8 (P4) I mean, I took my medication. I take everything by pill and that’s what I kept doing, every day the same thing. I didn’t think my medication [dose] would [need to] be reduced or anything in particular. The truth is that I didn’t really think very much
	Personal protective equipment & social distancing	5:14 (P5) Everyone was at their own home, and we saw each other every single day at 8 pm by videoconference and we saw each other [that way] until they said we could go out

### Anxiety, fear and vulnerability

The context of pandemic and confinement had a strong emotional impact on participants, and the most-expressed emotions were anxiety, fear, and vulnerability. Participants described the lockdown during first wave of the pandemic as something that was totally abnormal and hard to believe; they were shocked to hear the news of the number of deaths in Spain every day:

I thought I was dreaming. I thought this shouldn’t be happening in this day and age 3: 1 (P3).

One issue that respondents mentioned was the fear of infecting others, despite all the protective measures they used. For example, one participant, a health centre worker, was afraid of contagion in spite of the measures she took with her family:

In fact, at first I was worried that I might pass it on to them; I was working, I think the worst time was before [the state of emergency] (…). I got a room ready in case I had to isolate 5:13 (P5).

They also reported negative emotions, such as anxiety and worry:

I have anxiety problems, what`s been getting me down is the fact that I’m feeling a little agoraphobic 8:10 (P8).

It was the anguish of being locked away, of thinking you couldn’t see my 5-year-old granddaughter. My brother …. the family … my daughter and my son… 4:3 (P4).

Others felt fear at seeing so many COVID-19 infections at close range:

We’re all a bit scared. My children have all been through it, three of my four grandchildren. My daughter-in-law has had some awful aftereffects 3: 8 (P3).

Or at living close to death:

Scared. Because you see that the people who started to fall ill were mainly over 55 years old and it really hits you … 6: 1 (P6).

It was made worse by the experience of the loss of friends and family, or by news of acquaintances being admitted to the ICU:

I felt very sad to think of all the people who … I have relatives who have died and … it affected me a lot … not being able to be there … not being able to be with them 3.3 (P3).

On the other hand, some of them managed to keep these feelings of sadness at bay, thanks to their contacts with family, mainly through social media and video conferencing.

I saw them on the phone … and that kept me happy 7:16 (P7).

This feeling of social isolation was extremely negative:

I took it badly because I couldn’t leave the house, I couldn’t see my friends… 9.1 (P9).

For some, it was a negative experience because it disrupted their everyday routines and their self-care.

I felt terrible, it disrupted everything for me. I go to the pool for my water aerobics class, and everything was closed (…). I felt really bad having to spend all day at home 1: 1 (P1).

I used to walk two hours a day, when I was confined because I stayed at home, and I started to put on weight again … 6.4 (P6).

On the other hand, some respondents reported that the confinement and the change in their daily routines was an opportunity for reflection and thinking about their lives:

Three months, locked up at home without singing, without walking, without exercising … I mean, it practically gives you a vision of yourself, the experience of being alone for so many days, it’s a bit like being in a monastery (…). From this point of view the confinement was quite interesting 10.5 (P10).

### Insufficient diabetes monitoring by the health system

During the pandemic, health centres prioritised attention to COVID-19 patients, and on-site care of chronic diseases was postponed. Patients reported that their analyses and tests were cancelled:

During the pandemic no diabetes care was available. And even now, there are people who are being told over the phone that it isn’t important … they’re told not to come because no tests are being done 5.6 (P5).

Nonetheless, medication and supplies for diabetics were provided:

At the beginning of the pandemic, I went to look for supplies for diabetes and they gave me enough for three or four months 7.9 (P7).

Some respondents felt abandoned by the health staff who normally cared for them:

Abandoned… (silence) … The normal monthly check-up with the nurse to look at everything (…) didn’t happen. I also have blood tests every three or six months to check my sugar level… (…) but they didn’t happen either 8.2 (P8).

Some participants expressed not understanding the reason for the restriction:

Why can children go to school in a group, in a class, but a doctor can’t see you, they can only talk to you by phone … even though when you go for an appointment there’s a separation between you, the desk, you’re at least a yard away … and wearing their masks … and it turns out they can’t see you … well, a lack of personal protection … yes, you really notice it, because there has been a lot of neglect 8.5 (P8).

But others expressed more understanding of the situation even though they were not seen by health staff:

If you put yourself in their shoes, you realise they couldn't have done any more … 3.5 (P3).

Some patients realised that they had to take control of their disease, because no one else could help them; they ended up accepting the situation:

Well, you realise you’ve got to take care of yourself. And in all, a little self-discipline. Because I didn’t have anyone else to depend on, it was only me, there was no one else (P8).

Others stated that this situation did not affect them because they were already used to a patient-centred model and that the maximum responsibility for their care lay with them:

What sort of care do you expect? We have to care for ourselves … no matter how much they call me and ask me if I’m following my diet, if I’m eating properly, if I’m walking … no matter how much they call … it's up to you …. it's not an injury that you need someone to come and treat you, this is something that’s your own decision 4.5 (P4).

Most participants monitored their blood glucose:

Because I knew I had to check my glucose, I checked it every day and no problem 7.11 (P7).

### Proactive self-care

In the management of their disease during lockdown, patients with T2D introduced changes in terms of their physical activity, diet, and medication. Given the impossibility of going outside to exercise, many adapted their physical activity to their home space:

Well, being at home, I coped quite well. I went out onto the rooftop, where I was able to move around and pass the time. I walked up the stairs two or three times. So, I coped quite well 2.2 (P2).

Many participants established routines and did their regular activities, at different levels of intensity:

Every day, every day, every day, and it started … first I started 15 min a day, and then went up to 45 min every day and more intense; I walked fast, then I ran, faster and faster until I got a sore back 4.4 (P4).

This change in physical activity was regarded as a problem by some, but not by others:

I would open all the doors of the apartment and go around until I got tired, and when I got tired, I stopped. It was very boring 1: 5 (P1).

As there was time for everything (…), establishing a routine of walking one hour in the morning and one in the afternoon was not too hard 10: 7 (P10).

However, others abruptly stopped taking exercise:

I didn't do any physical activity while the stay-at-home order lasted 6.6 (P6).

All participants had access to fresh food and their normal diet, since the food shops stayed open during lockdown.

The shops where I go have got everything, fish, meat, chicken, everything 11.9 (P1).

Most reported good adherence to their regular diet:

Well, I saw that I couldn’t … do anything else, or go out … well, it's better to take care of yourself a little, isn’t it? This is also unconscious because I don't think about being diabetic … it’s something I’ve just accepted …. 13.7 (P4).

Others ate between meals, out of stress or boredom:

When I’m nervous, when I’m anxious … there are people whose stomachs close up, but I’m the opposite. I have snacks even though I’m not hungry 12.2 (P3).

None of the respondents had trouble getting their usual medication, and they followed their prescriptions, although they stressed that they were taking the medication without any medical supervision:

What I did is what I always did, there was no change. I went to the pharmacy every month to get my medication 11.9 (P1).

Most participants complied with the recommendations regarding personal protective equipment, hand washing, disinfection, ventilation of the home, and social distancing.

I was careful with my mask, I washed my hands a lot, and cleaned the flat 3.11 (P3).

Some participants applied specific protective measures in their homes:

At the door everyone took off their shoes, and they left their coats in a separate room, they sprayed their hands continuously, and every other day I changed the bed linen, ventilated the flat, cleaned everything. (…). Every time I went to the bathroom I pulled the chain with the lid down, and then cleaned my hands with disinfectant and the toilet as well 4:14 (P4).

Others reported taking particularly strict protective measures, due to their condition:

I took much more care (than other people) because I’m diabetic 8:12 (P8).

Some participants reported that they kept their distance from others, due to their diabetes:

I kept away because I thought I was much more likely to infect them than they were to infect me … so to avoid contagion I kept away from them 8:17 (P8).

Or that their families imposed this distancing on them, in order to protect them:

I asked her [the participant’s granddaughter] to give me a kiss because I needed one, but she said, “No, grandma, I have to go to school, and I don't want to” … And I said, “I’ll just give you a little kiss on your head” and she said “No, no, no!” She wouldn’t let me … 3: 9 (P3).

## Discussion

We have analysed the experiences of patients with T2D in lockdown during the first wave of the COVID-19 pandemic in Catalonia, Spain, and their strategies for managing their disease. Patients with diabetes felt especially vulnerable to infection, and presented emotional difficulties similar to those recorded in patients with COVID-19 at home or with other chronic conditions [6]. However, despite the changes they experienced in their daily lives and the barriers to accessing chronic care follow-up in primary care centres, they were able to establish routines for self-care.

### Fear, anxiety, and vulnerability

Global guidelines on containment measures for the prevention of COVID-19 place special emphasis on vulnerable populations, including people with diabetes [23]. Our results show that when patients were aware of the risk of contracting COVID-19 due to their T2D status they felt particularly vulnerable and fearful of falling ill. Our data are in line studies showing that having a chronic illness (including T2D), belonging to a risk group, or the death of a family member due to COVID-19 are positively associated with fear of COVID-19 [24]. The emotional impact of the pandemic was considerable, as the necessary lifestyle changes caused feelings of anxiety among many patients. Elsewhere, the pandemic has been associated with increased stress in general populations, and external stress may reduce physical activity and lead to a poorer diet [25].

The participants engaged in social distancing due to their fear of infecting others but found the experience to be emotionally challenging. Indeed, due to the high mortality related to COVID and the frequency of near-death experiences, an increased awareness of mortality has been reported during lockdown [25]. Not only diabetic patients have this perception: other patients with chronic and immunocompromised diseases such as cancer, rheumatoid arthritis, asthma, Crohn’s disease, hypertension, and cardiovascular disease also felt anxiety and fear during the pandemic [26].

Despite the negative emotional experience of most, for some participants, the suspension of everyday life routines represented an opportunity for reflecting on what was most important to them.

### Insufficient diabetes monitoring by the health system

In contrast to reports in other countries [27], our participants had no difficulty accessing medication or blood glucose control equipment such as glucose strips, needles, or glucometers. However, all of them encountered barriers to accessing primary care. Although they expressed understanding of the pandemic situation, many felt abandoned by the health care system, as other researchers have reported [28].

Our data suggest that the use of telemedicine and an e-Health model could achieve satisfactory levels of self-care especially in patients with an hb1Ac greater than 6. The popularisation of the Internet and the use of smartphones and emerging fifth-generation networks have allowed patients to attend medical appointments remotely instead of coming to the hospital during the COVID-19 outbreak.

Our results also provide relevant data regarding blood glucose control during the COVID-19 pandemic. Although diabetes is a primary risk factor for the development of severe and septic pneumonia due to infection, patients do not generally intensify their metabolic controls [29]. This may be due to a lack of information received from professionals monitoring the chronicity of primary pare or due to the lack of protocols or clinical practice guidelines adapted to the situation. In the study by [30], medication intake was significantly reduced during the pandemic, although in our study compliance with medication intake remained good.

Changes in the provision of health care due to the pandemic have created the need for greater attention to emotional and psychosocial health of patients with T2D. [31].

### Proactive self-care

The measures imposed by the authorities affected the daily life of the general population as well as that of patients with T2D. In general, this situation was experienced negatively, given that it caused social and family isolation.

The restrictions introduced by the authorities to prevent or reduce the risk of virus transmission led to significant changes in diabetes control. One of the nursing strategies applied to address the needs of patients with T2D in primary care was to promote self-care. Self-care-focused nursing interventions can achieve significant improvements in responsibility for health, physical activity, nutrition, and stress management [32].

All patients had access to fresh food, since food shops remained open during the lockdown and most patients continued with their usual diets. Most already had a good adherence to diet, although some reported eating between meals out of boredom. Although our participants had access to medication and food, the pandemic made it more difficult to manage their diabetes [31].

The results show that the stay-at-home order forced patients with T2D to limit their activities, including physical activity. Barone et al. [33] found that physical activity in diabetic patients fell by 59.5% during the COVID-19 pandemic and suggested that this variable be closely monitored due to its potential negative consequences on metabolic and cardiovascular health. As regards physical exercise, some patients reported decreased activity; others adapted their routines at home to be able to carry out physical activities recommended for a healthy lifestyle, such as walking, running, and going up and down stairs. A few participants performed no physical activity during lockdown, and some achieved optimal T2D risk prevention values, by brisk walking or by observing the current recommendation of 150 min/week of moderate aerobic activity or 30 min/day for 5 days/week [34]. Despite these changes in behaviour, however, the amount of time devoted to exercise was not optimal for preventing the risks caused by diabetes. The emotional and social impact on certain patients may also be related to the reduction in physical activity, as regular exercise is acknowledged to improve the mental and social health of patients with T2D [35].

### Limitations

This study has several limitations. The first is that the results can only be extrapolated to similar clinical contexts and similar users. The sample is small, and therefore is not representative of all T2D patients with a similar profile. This study can be a launch point, useful for comparison with larger studies in other contexts, to identify best practices in caring for people with T2D during a health crisis.

Second, the context in which the study was carried out was limited to primary care centres in Catalonia with specific sociodemographic characteristics and with good adherence to their prescribed T2D care. Including other types of patients from other geographic areas could provide different results.

Finally, our study includes only the perspective of patients. A fuller picture would emerge if the perspective of nurses monitoring diabetic patients were also included.

## Conclusions

This study has provided information on the experiences and emotional responses of patients with T2D during home confinement and on the adaptation of the management of their pathology in Catalonia, Spain. All participants were diabetic patients with good adherence to treatment prior to the pandemic. Due to their health status, patients reported feeling highly vulnerable and fearful of infection. Despite this, patients with T2D were able to establish self-care routines for physical activity and nutrition. In some cases, the lack of access to their normal care at primary care centres made them feel abandoned, although the fact that they were well and that their blood sugar levels were within the recommended levels meant that they did not feel particularly anxious; in general, they were sympathetic to the situation of the health workers. A silver lining of the pandemic may be the way it allowed patients to take control of their disease. This pro-activity on the part of patients should be considered in preparation for future health crises.

## Data Availability

The interviews were conducted by the lead author and she is the only researcher who knew the identity of the participants. Her record of interviewees’ names and other personal information will be deleted after publication. Data will be provided upon reasonable request.

## References

[1] Ioannidis JPA. OMS | Tasa de letalidad por la infección de la COVID-19 calculada a partir de los datos de seroprevalencia. WHO [Internet]. World Health Organization; 2021 [Cited 2021 May 26]; Available from: http://www.who.int/bulletin/volumes/99/1/20-265892-ab/es/

[2] Bellido V, Pérez A. Consequences of COVID-19 on people with diabetes. Endocrinol Diabetes y Nutr [Internet]. Elsevier Doyma; 2020 [cited 2021 May 26];67:355–6. Available from: https://www.elsevier.es/es-revista-endocrinologia-diabetes-nutricion-13-articulo-consecuencias-covid-19-sobre-personas-con-S253001642030104X10.1016/j.endinu.2020.04.001PMC721174832475769

[3] International Diabetes Federation. COVID-19: Perspectives from people with diabetes. Diabetes Res Clin Pract. 2020;163:108201. 10.1016/j.diabres.2020.108201. 10.1016/j.diabres.2020.108201 PMC7834891 32482296

[4] Banerjee M, Chakraborty S, Rimesh P. Diabetes self-management amid COVID-19 pandemic | Elsevier Enhanced Reader. Diabetes Metab Syndr [Internet]. 2020;14:351–4. [Cited 2021 May 26]. Available from: https://reader.elsevier.com/reader/sd/pii/S1871402120300783?token=FB54D3A227BBCC2E2790E384834A32E17A24F9748CDB1F68563AA3F7178AD32A01D7F49F8B1A42EF80BD4D2E9DEB83B3&originRegion=eu-west-1&originCreation=20210526105736. 10.1016/j.dsx.2020.04.013 PMC7194953 32311652

[5] Schmidt SK, Hemmestad L, Macdonald CS, Langberg H, Valentiner LS (2020). Motivation and barriers to maintaining lifestyle changes in patients with type 2 diabetes after an intensive lifestyle intervention (The U-TURN trial): A longitudinal qualitative study. Int J Env Res Public Heal..

[6] Musche V, Kohler H, Bäuerle A, Schweda A, Weismüller B, Fink M, et al. Covid-19-related fear, risk perception, and safety behavior in individuals with diabetes. Healthc [Internet]. 2021;9:480. MDPI AG; [Cited 2021 Jul 19]. Available from: 10.3390/healthcare9040480. 10.3390/healthcare9040480 PMC8072870 33919519

[7] Sánchez-Villena AR, de La Fuente-Figuerola V. COVID-19: cuarentena, aislamiento, distanciamiento social y confinamiento, ¿son lo mismo? An Pediatr [Internet]. 2020;93:73–4. Elsevier; [Cited 2022 Mar 1]. Available from: https://www.analesdepediatria.org/es-covid-19-cuarentena-aislamiento-distanciamiento-social-articulo-S1695403320301776. 10.1016/j.anpedi.2020.05.001 PMC7211640 32507611

[8] Eberle C, Stichling S. Impact of COVID-19 lockdown on glycemic control in patients with type 1 and type 2 diabetes mellitus: a systematic review. Diabetol Metab Syndr [Internet]. BioMed Central Ltd. 2021;13:1–8. [Cited 2022 Mar 5]. Available from: 10.1186/s13098-021-00705-9. 10.1186/s13098-021-00705-9 PMC8423337 34493317

[9] Makki I, Alnoon N, Rahmani N, Almulla J, Alamiri A, Alfalasi A, et al. Impact of COVID 19 Lockdown on Glycemic Control in Patients with Type 2 Diabetes Mellitus in Dubai. Curr Diabetes Rev [Internet]. Curr Diabetes Rev; 2021 [cited 2022 Mar 5];18. Available from: https://pubmed.ncbi.nlm.nih.gov/34879807/10.2174/1573399818666211208102729 34879807

[10] Kemppainen V, Tossavainen K, Turunen H (2013). Nurses’ roles in health promotion practice: An integrative review. Heal Promot Int.

[11] Agencia de SalutPública de Catalunya (2020). INFORME-TECNIC-1-COVID-19–19032020.

[12] Ojeda Ibáñez MF. Telemedicina como estrategia para el control de los pacientes con diabetes mellitus tipo ll en el contexto de pandemia por la COVID-19. Estado del arte [Internet]. Universidad Peruana Cayetano Heredia; 2021. Available from: https://repositorio.upch.edu.pe/handle/20.500.12866/9262

[13] Zhai YK, Zhu WJ, Cai YL, Sun DX, Zhao J. Clinical- and cost-effectiveness of telemedicine in type 2 diabetes mellitus: a systematic review and meta-analysis. Medicine (Baltimore) [Internet]. Medicine (Baltimore); 2014 [cited 2022 Mar 1];93:e312. Available from: https://pubmed.ncbi.nlm.nih.gov/25526482/10.1097/MD.0000000000000312 PMC4603080 25526482

[14] Singh K, Kaushik A, Johnson L, Jaganathan S, Jarhyan P, Deepa M, et al. Patient experiences and perceptions of chronic disease care during the COVID-19 pandemic in India: a qualitative study. BMJ Open [Internet]. BMJ Open; 2021 [cited 2022 Mar 2];11. Available from: https://pubmed.ncbi.nlm.nih.gov/34145019/10.1136/bmjopen-2021-048926 PMC8214993 34145019

[15] Shi C, Zhu H, Liu J, Zhou J, Tang W. Barriers to self-management of type 2 diabetes during covid-19 medical isolation: A qualitative study. Diabetes Metab Syndr Obes [Internet]. 2020;13:3713–25. Dove Medical Press Ltd; [Cited 2021 Jul 22]. Available from: https://pubmed.ncbi.nlm.nih.gov/33116721/. 10.2147/DMSO.S268481 PMC7569039 33116721

[16] Colorafi KJ, Evans B. Qualitative Descriptive Methods in Health Science Research. HERD [Internet]. 2016;9:16–25. HERD; [Cited 2022 Mar 7]. Available from: https://pubmed.ncbi.nlm.nih.gov/26791375/. 10.1177/1937586715614171 PMC7586301 26791375

[17] Morse JM. Molding qualitative health research. Qual Heal Res [Internet]. SAGE PublicationsSage CA: Los Angeles, CA; 2011 [cited 2022 Mar 7];21:1019–21. Available from: https://journals.sagepub.com/doi/10.1177/1049732311404706 10.1177/1049732311404706 21768617

[18] Tulloch H, Sweet S, Fortier M, Capstick G, Kenny G, Sigal R. Exercise facilitators and barriers from adoption to maintenance in the diabetes aerobic and resistance exercise trial. Can J Diabetes [Internet]. 2013;37:367–74. [Cited 2014 Dec 17]. Available from: http://www.sciencedirect.com/science/article/pii/S1499267113012744. 10.1016/j.jcjd.2013.09.002 24321716

[19] Saunders B, Sim J, Kingstone T, Baker S, Waterfield J, Bartlam B, et al. Saturation in qualitative research: exploring its conceptualization and operationalization. Qual Quant [Internet]. 2018;52:1893–907. Qual Quant; [Cited 2022 Mar 15]. Available from: https://pubmed.ncbi.nlm.nih.gov/29937585/. 10.1007/s11135-017-0574-8 PMC5993836 29937585

[20] Braun V, Clarke V (2014). What can ‘thematic analysis’ offer health and wellbeing researchers?. Int J Qual Stud Health Well-being.

[21] Guba E, Lincoln Y, Denzin N, Lincoln Y (1994). Competing paradigms in qualitative research. Handb Qual Res.

[22] Tong A, Sainsbury P, Craig J (2007). Consolidated criteria for reporting qualitative research (COREQ): A 32-item checklist for interviews and focus groups. Int J Qual Heal Care.

[23] Beran D, Aebischer Perone S, Castellsague Perolini M, Chappuis F, Chopard P, Haller DM (2021). Beyond the virus: Ensuring continuity of care for people with diabetes during COVID-19. Prim Care Diabetes.

[24] TzurBitan D, Grossman-Giron A, Bloch Y, Mayer Y, Shiffman N, Mendlovic S (2020). Fear of COVID-19 scale: Psychometric characteristics, reliability and validity in the Israeli population. Psychiatry Res..

[25] Munekawa C, Hosomi Y, Hashimoto Y, Okamura T, Takahashi F, Kawano R, et al. Effect of coronavirus disease 2019 pandemic on the lifestyle and glycemic control in patients with type 2 diabetes: A cross-section and retrospective cohort study. Endocr J [Internet]. 2021;68:201–10. Japan Endocrine Society; [Cited 2021 Jul 21]. Available from: https://pubmed.ncbi.nlm.nih.gov/32999133/. 10.1507/endocrj.EJ20-0426 32999133

[26] Al-Rahimi JS, Nass NM, Hassoubah SA, Wazqar DY, Alamoudi SA. Levels and predictors of fear and health anxiety during the current outbreak of COVID-19 in immunocompromised and chronic disease patients in Saudi Arabia: A cross-sectional correlational study. PLoS One [Internet]. Public Library of Science; 2021 [cited 2021 Jul 22];16. Available from: https://pubmed.ncbi.nlm.nih.gov/33901260/10.1371/journal.pone.0250554 PMC8075243 33901260

[27] Wicaksana AL, Hertanti NS, Ferdiana A, Pramono RB. Diabetes management and specific considerations for patients with diabetes during coronavirus diseases pandemic: A scoping review. Diabetes Metab Syndr. 2020;14(5):1109–20. 10.1016/j.dsx.2020.06.070. 10.1016/j.dsx.2020.06.070 PMC7334970 32659694

[28] Jeong IK, Yoon KH, Lee MK (2020). Diabetes and COVID-19: Global and regional perspectives. Diabetes Res Clin Pr..

[29] Bornstein SR, Rubino F, Khunti K, Mingrone G, Hopkins D, Birkenfeld AL (2020). Practical recommendations for the management of diabetes in patients with COVID-19. Lancet Diabetes Endocrinol Elsevier Ltd.

[30] Alshareef R, Zahrani A Al, Alzahrani A, Ghandoura L. Impact of the COVID-19 lockdown on diabetes patients in Jeddah, Saudi Arabia. Diabetes Metab Syndr Clin Res Rev [Internet]. 2020;14:1583–7. Elsevier Ltd; [Cited 2021 May 26]. Available from: https://pubmed.ncbi.nlm.nih.gov/32947759/10.1016/j.dsx.2020.07.051 PMC7422800 32947759

[31] Fisher L, Polonsky W, Asuni A, Jolly Y, Hessler D. The early impact of the COVID-19 pandemic on adults with type 1 or type 2 diabetes: A national cohort study. J Diabetes Complicat [Internet]. J Diabetes Complications; 2020 [cited 2022 Mar 15];34. Available from: https://pubmed.ncbi.nlm.nih.gov/33059981/10.1016/j.jdiacomp.2020.107748 PMC7539933 33059981

[32] Spoorenberg S, Wynia K, Uittenbroek RJ, Kremer H, Reijneveld SA. Effects of a population-based, person-centred and integrated care service on health, wellbeing and self-management of community-living older adults: A randomised controlled trial on Embrace. PLoS One. 2018;13. 10.1371/journal.pone. 10.1371/journal.pone.0190751 PMC5774687 29351295

[33] Barone MTU, Harnik SB, de Luca PV, Lima BL de S, Wieselberg RJP, Ngongo B, et al. The impact of COVID-19 on people with diabetes in Brazil. Diabetes Res Clin Pr. 2020;166:108304. 10.1016/j.diabres.2020.108304. 10.1016/j.diabres.2020.108304 PMC7332443 32623040

[34] Colberg SR, Sigal RJ, Fernhall B, Regensteiner JG, Blissmer BJ, Rubin RR, et al. Exercise and type 2 diabetes: The American College of Sports Medicine and the American Diabetes Association: Joint position statement. Diabetes Care. 2010;33(12):e147–67. 10.2337/dc10-9990. 10.2337/dc10-9990 PMC2992225 21115758

[35] Wylie TAF, Shah C, Connor R, Farmer AJ, Ismail K, Millar B (2019). Transforming mental well-being for people with diabetes: research recommendations from Diabetes UK’s 2019 Diabetes and Mental Well-Being Workshop. Diabet Med.

